# Supplementary material for: MUG CCArly: A Novel Autologous 3D Cholangiocarcinoma Model Presents an Increased Angiogenic Potential
Source: Cancers (Basel). 2023 Mar 14;15(6):1757. doi: 10.3390/cancers15061757 (PMC10046314; doi:10.3390/cancers15061757)
Supplement: Supplementary file 1 [file cancers-15-01757-s001.zip › cancers-2278866-supplementary material.pdf]

**Table S1.** Antibodies used for IHC and ICC staining.

| Antibody | Species | Dilution | Distributor |
|---|---|---|---|
| Cytokeratin 7 | mouse | 1:100 | Dako (Santa Klara, USA) |
| Cytokeratin 19 | mouse | 1:100 | Dako (Santa Klara, USA) |
| E-cadherin | mouse | ready to use | Dako (Santa Klara, USA) |
| Vimentin | mouse | ready to use | Linaris (Dossenheim, Germany) |
| Tenascin C | mouse | 1:100 | Origene Technologies (Maryland, USA) |
| PDGFR beta | rabbit | 1:100 | Lab Vision / Neomarkers (CA, USA) |
| $\alpha$-SMA | mouse | 1:100 | Dako (Santa Klara, USA) |

**Table S2.** STR authentication of MUG CCArly and CCArly CAF. STR profile for 16 loci are illustrated for the primary tumor tissue and the corresponding cell lines.

| STR-Locus | D3S1358 | TH01 | D21S11 | D18S51 | Penta E | D5S818 | D13S317 | D7S820 |
|---|---|---|---|---|---|---|---|---|
| Tumor tissue | 16, 16 | 8, 9.3 | 28, 29 | 16, 16 | 12, 13 | 10, 12 | 8, 9 | 10, 12 |
| MUG CCArly p17, p36 | 16, 16 | 8, 9.3 | 28, 29 | 16, 16 | 12, 13 | 10, 12 | 8, 9 | 10, 13 |
| CCArly CAFs p7, p33 | 16, 16 | 8, 9.3 | 28, 29 | 16, 16 | 12, 13 | 10, 12 | 8, 9 | 10, 12 |

| STR-Locus | D16S539 | CSF1PO | Penta D | Amelogenin | vWA | D8S1179 | TPOX | FGA |
|---|---|---|---|---|---|---|---|---|
| Tumor tissue | 11,14 | 11, 11 | 10, 11 | X, X | 16, 19 | 13, 13 | 8, 10 | 20, 22 |
| MUG CCArly p17, p36 | 11,14 | 11, 11 | 11, 11 | X, X | 16, 19 | 13, 13 | 8, 10 | 20, 22 |
| CCArly CAFs p7, p33 | 11,14 | 11, 11 | 10, 11 | X, X | 16, 19 | 13, 13 | 8, 10 | 20, 22 |

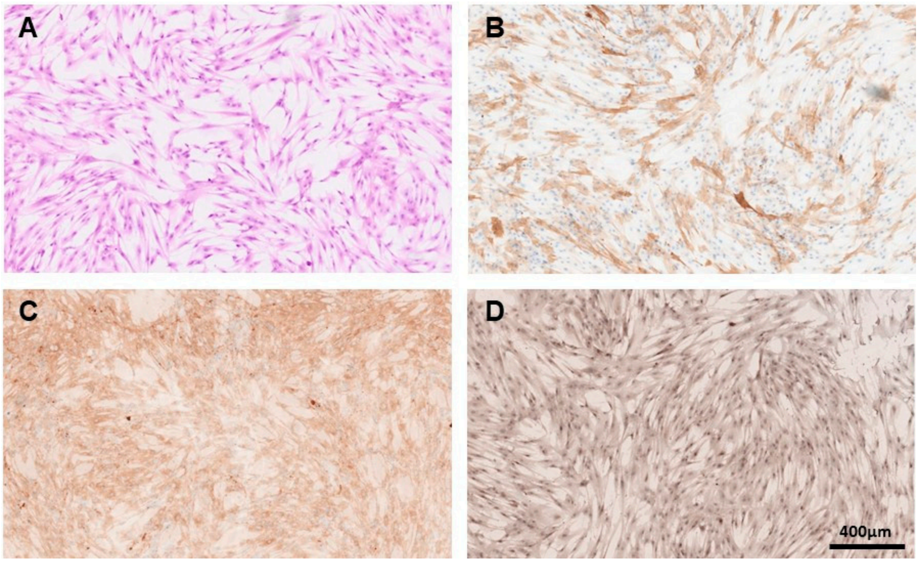


**Figure S1.** IHC staining of CCArly CAF for CAF markers. **A**: H&E, **B**: $\alpha$-SMA, **C**: TNC, **D**: PDGFRB. Scale bar: 400 µm.

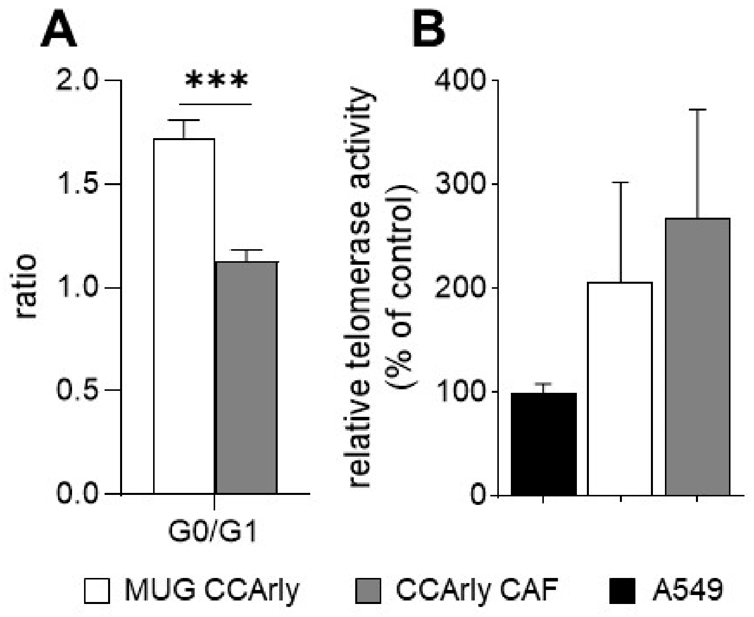


**Figure S2.** DNA ploidy index of MUG CCArly and CCArly CAF and relative telomerase activity. **A**: DNA index ratios are presented as comparison to diploidic PBMCs. **B**: The relative telomerase activity was normalized to a positive control cell line A549. $n = 3$, bars represent mean ± SEM. * $p > 0.05$, ** $p > 0.01$, *** $p < 0.001$. Statistical test: two-tailed $t$-test.

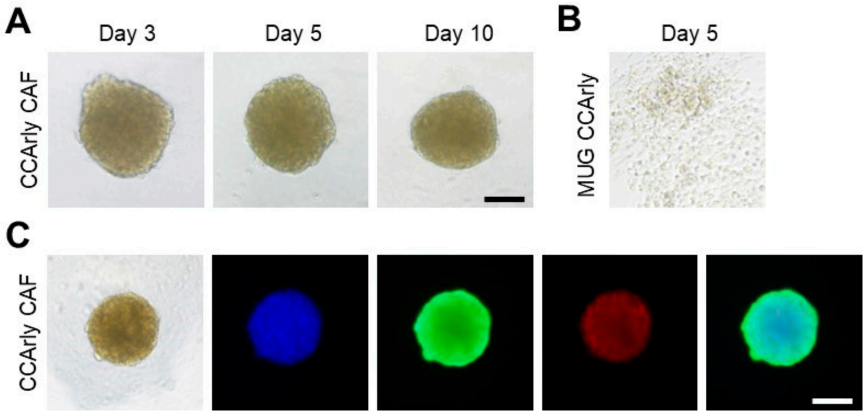


**Figure S3.** Single-cultured CCArly CAF and MUG CCArly spheroids. **A**: CCArly CAF spheroid formation over time under the bright field microscope. **B**: Single cells of MUG CCArly on day five without collagen supplementation. **C**: Live / dead fluorescently stained CCArly CAF spheroids on day nine. Hoechst 33342, cell nuclei; Calcein AM, viable cells; Ethidium homodimer-I, dead cells; merge, all three dyes merged. BF, bright field; scale bar, 100 μm.

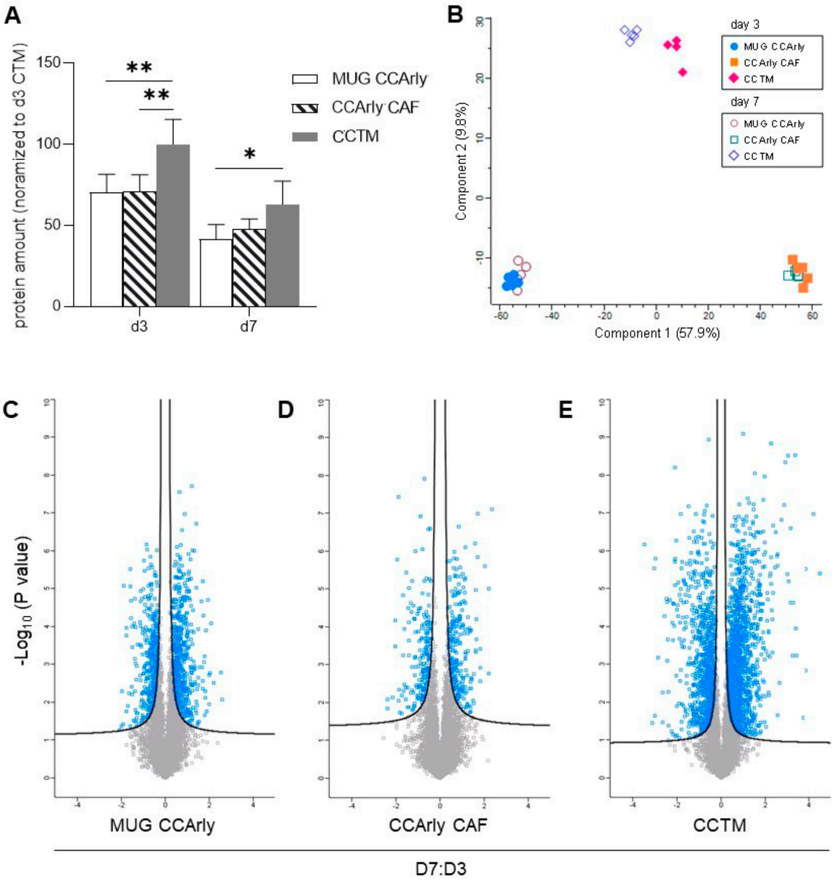


**Figure S4.** Quality control of proteomic analysis. A: Protein concentration measurement using the DC assay was normalized to total protein concentrations of the CCTM group on day 3. B: Principal component analysis (PCA) showing all biological replicates of all three groups and both measured time points. C: Volcano blots comparing proteins of day three and day seven for all three groups. *n* = 5.
